# Home visits as an interprofessional learning activity for students in primary healthcare

**DOI:** 10.1017/S1463423620000572

**Published:** 2020-12-10

**Authors:** Eva Toth-Pal, Cecilia Fridén, Stefano Torres Asenjo, Christina B. Olsson

**Affiliations:** 1 Academic Primary Healthcare Centre, Region Stockholm, Stockholm, Sweden; 2 Department of Neurobiology, Care Sciences and Society, Division of Family Medicine and Primary Care, Karolinska Institutet, Stockholm, Sweden; 3 Department of Neurobiology, Care Sciences and Society, Division of Physiotherapy, Karolinska Institutet, Stockholm, Sweden; 4 Department of Neurobiology, Care Sciences and Society, Karolinska Institutet, Stockholm, Sweden

**Keywords:** education, house calls, teamwork, undergraduate

## Abstract

**Aim:**

To evaluate person-centred home visits as an interprofessional learning (IPL) activity for undergraduate students during clinical placements in primary healthcare.

**Background:**

Interprofessional collaboration is known to improve patient safety, increase job satisfaction, and reduce stress among healthcare professionals. Students should already during their basic training experience interprofessional collaboration.

**Methods:**

Students from six different educational programmes and supervisors and adjunct clinical lecturers from different professions participated in the learning activity. The students read a description of the patient history before the visit together with a supervisor. During the home visit, the students were responsible for history-taking and for performing relevant examinations. Afterwards, the students made a joint care plan for the patient. Students, supervisors, and adjunct clinical lecturers discussed the outcomes in a seminar and reflected on each other’s professional roles. The students and the patients answered a questionnaire about the activity, and the supervisors and the adjunct clinical lecturers were interviewed in focus groups.

**Findings:**

Thirty interprofessional home visits were conducted, involving 109 students from six different healthcare professions. The students reported that they had gained insights into how different professions could collaborate and an increased understanding of teamwork. All patients were satisfied with the visits and felt that they had been listened to. The interview analysis showed one overarching theme: ‘*Interprofessional home visits in primary healthcare were an appreciated and effective pedagogical learning activity with a sustainability dependent on organisational factors’*.

**Conclusions:**

The students felt that participation in the activity increased their understanding of collaboration and of other professions’ skills. The supervisors found the home visits to be an appreciated and effective learning activity. The results indicate that this learning activity can be used in primary healthcare settings to promote students’ IPL, but organisational factors need to be considered in order to support sustainability.

## Introduction

Health services are entering a new era in most Western countries as a consequence of the rapid change in demographics that is underway. Populations are ageing, which means that more people will live longer with chronic diseases, and these account for a large part of healthcare costs. To meet these challenges, healthcare needs to shift its focus from acute hospital care towards a more chronic care model, centred in primary healthcare. Patients with chronic conditions often require the competence of more than one profession for their care, and in many cases require regular and lifelong contact with healthcare. Healthcare systems must develop new working methods to meet these needs (Bodenheimer *et al*., 2002; Thistlethwaite, 2012).

Interprofessional collaboration is based on shared decision making and shared responsibility, where the perspective of all professions is taken into account, and the patient and their family members are an integral part of the team (Petri, 2010). Interprofessional collaboration has been shown to lead to more efficient care (Petri, 2010).

To facilitate interprofessional collaboration, students need to learn this approach already during their basic training. According to the Centre for the Advancement of Interprofessional Education (CAIPE), interprofessional education is ‘*when two or more professions learn with, from, and about each other to improve collaboration and quality of care’* (CAIPE, 2002). According to the World Health Organization (WHO) (Gilbert *et al*., 2010), interprofessional education is also an important method for meeting the growing shortage of healthcare professionals.

Traditionally, different healthcare professionals are trained separately with little interaction during their education. New graduates are expected to be able to collaborate with other staff without the knowledge of other professions’ competencies (Thistlethwaite, 2012). Interprofessional education is important for overcoming the inherent disadvantages of silo-based training models and can help students to acquire appropriate knowledge, skills, and attitudes. They should learn to take responsibility and use their expertise in collaboration with others in a way that will benefit the patient, already during their basic training (Thistlethwaite, 2012).

According to a review by Thistlethwaite (2012), students need to identify with their own profession in order to be able to participate effectively in interprofessional educational activities. Most healthcare students are able to distinguish their profession from other professions early in their training, which indicates that interprofessional education can be used early in the educational programmes (Thistlethwaite, 2012). Interprofessional educational activities need to simulate real care situations in order to be more effective, and lack of authenticity could be a problem (Thistlethwaite, 2012; Miller *et al.*, 2019).

Most research concerning interprofessional work and education has been performed in hospital environments (Drummond *et al.*, 2012; Tran *et al*., 2018). It has previously been noted that collaboration in hospitals is generally easier because the majority of the professions are located in the same facilities (Tran *et al*., 2018). Interprofessional working methods are not yet equally implemented in primary care, and consequently interprofessional learning (IPL) has been studied less frequently (Tran *et al*., 2018). The shift to a more community-based healthcare requires development of team-based care models that are suitable for both primary care practitioners and students (Thistlethwaite, 2012). One good example is the Leicester model (Anderson and Lennox, 2009), which has been developed over the course of many years of implementing IPL in community-based care. The model involves students from many health disciplines. Evaluations of the model have shown positive results, and it has become a part of each professional curriculum. Development of IPL in primary healthcare in Sweden only started recently, although many health educational programmes already incorporated intended learning outcomes for IPL.

The aim of this project was to implement and evaluate person-centred home visits as an educational model of IPL in primary healthcare during healthcare students’ clinical practice.

## Materials and methods

Interprofessional home visits were organised during students’ clinical placements in primary healthcare with a subsequent seminar led by adjunct clinical lecturers and supervisors. We used a mixed methods approach for evaluation in order to gather the perspectives of all participants. Students and patients were asked to answer a questionnaire, while adjunct clinical lecturers and supervisors were invited to participate in focus group interviews. A questionnaire was considered suitable for data collection from the students since they only participated in the activity once, and it would not have been feasible to interview all students.

### The interprofessional learning activity

The IPL activity was divided into three parts: i) the home visit, ii) students working out a care plan for the patient, and iii) a seminar including discussion of the case and reflection about the roles of different healthcare professionals. The interprofessional home visits were planned by the adjunct clinical lecturers along with the supervisors at the participating healthcare units. Three or four students from different educational programmes were invited to participate in each home visit. Each student group performed one home visit. Prior to the home visit the students read a short description about the patient case and decided who would be responsible for what during the visit. A supervisor accompanied them during the visit, but the students were responsible for history-taking and for performing relevant examinations. The average duration of home visits was one hour. After the home visit, the students discussed the case, made a joint report of their findings, and devised a care plan for the patient. Thereafter, the students presented the care plan and their reflections about the patient case in a discussion seminar with all students, supervisors, and adjunct clinical lecturers. The students’ care plan was then handed over to the patients’ primary caregivers for further decisions. Each student’s profession was represented by a supervisor during the reflection seminar regarding the patient’s care plan and the different professional roles. The seminar lasted about one hour, and the total time for the learning activity was about three hours. Between September 2016 and June 2018, a total of 30 home visits and subsequent seminars were conducted.

### Participants

Six primary healthcare units in three different parts of the Stockholm area participated in the study. Students from six different professions were recruited by the adjunct clinical lecturers and supervisors in each unit. The student professions were medical, nursing, physiotherapy, occupational therapy, speech therapy, and dietician. Patients were selected and invited by the primary caregiver, most often by staff working in home care. All participants consented to be part of the study, and the study was approved by the Regional Ethical Board in Stockholm, Sweden (2016/1025–31).

### Student and patient questionnaire

The student questionnaire contained eight questions on a 10-point Likert scale, and there was space for free-text comments at the end. The questions are presented in Table 1. Four questions assessed whether the students perceived that they had increased insight regarding their own and others’ professional competences after the learning activity, and four questions assessed whether the student teams felt they had succeeded in taking into account all of the patient’s needs. Students were also asked to indicate their profession. The patient questionnaire contained four questions on a 4-point Likert scale along with three open-ended questions for free-text responses about the patient’s experience of the home visit.

**Table 1:** Student questionnaire. Questions were answered on a 10-graded Likert scale. Questions 1–4 were about whether students perceived increased insight in one’s own and others’ professional competences after the learning activity. Questions 5–8 were about whether the student teams felt that they succeeded in taking care of all the patient’s needs. Question 9 was about whether students perceived the supervision had been a support during the learning activity.

	Student category														
	Total		Medical		Nursing		Physiotherapy		Occupational therapy		Speech therapy		Dietician		
Question	*N*	Median (min, max)	*N*	Median (min, max)	*N*	Median (min, max)	*N*	Median (min, max)	*N*	Median (min, max)	*N*	Median (min, max)	*N*	Median (min, max)	*P*-value*
Q1. Insight into how different professions can work together.	106	9 (4, 10)	34	8 (5, 10)	38	9 (4, 10)	19	9 (7, 10)	10	9.5 (6, 10)	4	10 (8, 10)	1	7 (7, 7)	0.287
Q2. Insight into my own professional role.	106	8 (3, 10)	34	7 (3, 10)	38	8.5 (5, 10)	19	8 (5, 10)	10	9 (5, 10)	4	9.5 (7, 10)	1	8 (8, 8)	0.012**
Q3. Understanding of competencies of the other professions.	106	9 (3, 10)	34	9 (5, 10)	38	8 (3, 10)	19	9 (4, 10)	10	9 (7, 10)	4	9 (8, 10)	1	9 (9, 9)	0.415
Q4. Understanding of my own role in teamwork.	106	8 (3, 10)	34	7.5 (4, 10)	38	8 (3, 10)	19	9 (7, 10)	10	9 (8, 10)	4	9 (8, 10)	1	9 (9, 9)	0.030**
Q5. Understanding of teamwork.	103	9 (3, 10)	33	8 (3, 10)	36	9 (5, 10)	19	8 (4, 10)	10	9.5 (7, 10)	4	9 (6, 10)	1	7 (7, 7)	0.592
Q6. Attention to the patient’s need for medical treatment.	102	9 (2, 10)	33	9 (2, 10)	36	9 (4, 10)	18	10 (8, 10)	10	9.5 (8, 10)	4	10 (9, 10)	1	4 (4, 4)	0.024**
Q7. Attention to the patient’s need for nursing.	102	9 (4, 10)	33	9 (6, 10)	36	9 (5, 10)	18	9.5 (4, 10)	10	9 (6, 10)	4	9 (9, 10)	1	8 (8, 8)	0.420
Q8. Attention to the patient’s need for rehabilitation.	102	9 (2, 10)	33	9 (6, 10)	36	9 (4, 10)	18	10 (7, 10)	10	10 (2, 10)	4	10 (8, 10)	1	9 (9, 9)	0.580
Q9. Supervision has been a support during the activity.	99	9 (4, 10)	31	9 (4, 10)	35	9 (5, 10)	18	9.5 (5, 10)	10	10 (5, 10)	4	10 (8, 10)	1	9 (9, 9)	0.678

Q = question.

* Kruskal-Wallis test.

** *P* < 0.05.

### Focus group interviews

Semi-structured focus group interviews were conducted with all the adjunct clinical lecturers and supervisors who participated in the home visits and/or in the subsequent seminars. The purpose of the interviews was to gain insight into the learning process during the IPL activity, as well as facilitators and barriers to the implementation of home visits as a routine IPL. The interviews were conducted at the end of each semester. Adjunct clinical lecturers and supervisors participated in separate focus group interviews. In total, four focus group interviews were conducted with adjunct clinical lecturers with 5–8 participants in each group. There were five interviews conducted with supervisors with 2–5 participants each. In addition, two individual interviews were conducted with adjunct clinical lecturers who could not participate in the focus groups. Since the study lasted for two years, some individuals were interviewed on more than one occasion. For each interview, one of the researchers took on the role of facilitator (ETP or CO), and one of the others took on the role of observer. A semi-structured interview guide was used (see Attachment). The observer noted bullet points on a whiteboard during the interviews, and the participants were asked at the end of each interview whether this summary reflected what had been said. All interviews were audio-recorded and subsequently transcribed verbatim. During the study period, the researchers had ongoing cooperation with the adjunct clinical lecturers in other interprofessional student activities outside this project, but there was no dependency in their relationships.

### Data analysis

Descriptive statistics were used for analysis of the student questionnaires, and differences between professions were calculated using the Kruskal–Wallis test. Interview data were analysed using qualitative content analysis (Graneheim and Lundman, 2004). The transcribed interviews were read through in their entirety. Meaning units were identified and condensed, and the condensed units were coded and then organised into sub-categories and categories. The subcategories and categories were finally abstracted to an overarching theme symbolising the latent content (Graneheim and Lundman, 2004).

## Results

### Student questionnaires

A total of 109 students from six different healthcare professions participated in the activity and all responded to the questionnaire. The number and distribution in terms of profession and sex was *n* (male/female) – medical: 34 (20/14), nursing: 39 (5/33), physiotherapy: 19 (5/14), occupational therapy: 11 (2/8), speech therapy: 4 (0/4), and dietician: 1 (0/1). Three students did not answer the question about their sex, and one did not answer the question about their profession.

Overall the students felt that they had gained insight into how different professions in primary healthcare could collaborate, an increased understanding of teamwork, and also insight into the other professions’ areas of competence (Table 1). There were significant differences between professions for three of the questions (nos. 2, 4, and 6). Medical students perceived that they had gained insight into their future professional role and into their own role in teamwork to a lesser extent than students from other professions reported. Medical and nursing students did not perceive that the patient’s medical needs had been noted to the same extent as other professions did.

### Patient questionnaires

In total, 30 patients participated in the home visits and 29 completed the questionnaire. All responding patients were very satisfied with the visits and felt that they had been listened to and treated with respect by the students. Two patients felt that there were too many students at the same time, though they were satisfied with the visit otherwise.

### Focus group interviews

A total of 22 adjunct clinical lecturers and supervisors participated in the interviews. They represented different professions (seven physiotherapists, six district nurses, five general practitioners, two occupational therapists, one dietician, and one speech therapist). Background data were collected on age, number of years working in the profession, number of years as an adjunct clinical lecturer or supervisor, number of years working in primary healthcare, previous training in IPL, and whether they had previously supervised IPL activities (Table 2). Five of the participants did not complete the background questionnaire.

**Table 2.** Background data for adjunct clinical lecturers (*n* = 8) and supervisors (*n* = 9)

Participants (*N* = 17)	
Age (years), median (min-max)	47 (29–64)
Male/female (*n*)	3/14
Number of years in the profession, median (min-max)	15 (4–34)
Number of years as an adjunct clinical lecturer, median (min-max)	3 (0.5–11)
Number of years as a supervisor, median (min-max)	7.5 (0.5–28)
Number of years in primary healthcare, median (min-max)	9 (0.25–28)
Some education in IPL (course, seminar, other), *n* (%)	12 (70.5%)
Experience of supervising IPL activities, *n* (%)	6 (35.3%)

We identified five categories and 19 subcategories in the qualitative content analysis. The overarching theme was ‘Interprofessional home visits in primary healthcare were an appreciated and effective pedagogical learning activity with a sustainability dependent on organisational factors’. The theme, main categories, and subcategories are presented in Table 3.

**Table 3.** The overarching theme, categories, and subcategories from the focus group interviews with adjunct clinical lecturers and supervisors.

Overarching theme	Categories	Sub-categories
Interprofessional home visits in primary healthcare were an appreciated and effective pedagogical learning activity with a sustainability dependent on organisational factors	Home visits as an educational IPL activity	– Student activating in an effective way – Instructive IPL activity when many professions participated – The supervisor was a facilitator, which can be a new role for some supervisors
	The activity was highly valued by the participants	– The students developed both in their professional and in their interprofessional roles – The supervisors experienced the activity as instructive and beneficial for patient care – Interprofessional assessments led to quality improvement for the patient
	Facilitating factors and challenges for the learning activity	– Difficult for supervisors to allocate time for the seminars – To organise the activity required extensive planning, but there were tricks to facilitate it – Finding the right patient was a key factor, but it turned out to be difficult – Designating sufficient time for organising the activity was perceived as a prerequisite
	Organisational prerequisites for IPL and teamwork in primary healthcare	– Separate organisations and geographical distances made cooperation more difficult – The financial compensation for supervision was less than the reimbursement for care visits – Support from the local management could make a big difference – More engagement from and better co-planning with health education programmes would facilitate IPL – Experience of interprofessional collaboration in the present clinical work varied
	Opportunities for dissemination	– The learning activity must be simplified if more students are to be able to participate – If there is to be dissemination, supervisors must be more involved – The project has opened for increased interprofessional collaboration in some cases – Support from the adjunct clinical lecturers was important

### Description of categories

1. Home visits as an educational IPL activity

The interviewed supervisors and adjunct clinical lecturers described how the students worked autonomously when they led the home visits themselves and how they took responsibility for the visits. The students supported each other in their tasks. The supervisor was mostly in the background, providing some support when appropriate. During the home visits, the students had their focus on the patient and showed consideration and respect both to the patient and to each other. Later, at the seminars, the students led and actively participated in the discussions.

The adjunct clinical lecturers expressed the importance of all professions’ supervisors participating in the seminar because the professions complemented each other in the discussions. The seminar broadened the students’ perspectives on teamwork and helped them see the multi-dimensional view in care.

The supervisor as a facilitator had to think about the needs of all student professions and ensure that all students were active. The supervisors perceived that working in this interprofessional context felt like a more genuine and modern form of pedagogy.

‘When it [the home visit] is interprofessional and there are students from several professions, then it is their home visit … they support each other.’ (supervisor)

‘We can see that this interprofessional thinking is beneficial, it broadens their horizons.’ (adjunct clinical lecturer)

‘When I am supervising interprofessionally, I feel much more that I am a real supervisor; there is more genuine pedagogy in that role.’ (adjunct clinical lecturer)

1. The activity was highly valued by the participants

The interviewees described how the students found it positive to work together and to discuss the visits with students from other professions. Students gained insight into what the different professions do and who could solve different problems. The students discovered the value of collaboration and found that they needed each other’s skills to be able to help the patient.

Some supervisors were hesitant to participate in the activity in the beginning. After participating they became very positive, as they experienced the seminar to be instructive and thought it was rewarding to meet students from other professions.

The interviewees reported that the students found new information during the IPL activity and that the discussion gave new insights, which led to an improvement in the quality of the patient’s care. The patients appreciated being examined by students from several different professions and having their medical problems considered from a variety of perspectives.

‘He actually understood that the competency that the others [students] have is of another kind, which he does not have … that he needs.’ (supervisor)

‘In fact, I learn something new each time … either from the students or from the supervisors who support the discussions. They very often add some new information; it is amusing, really fun.’ (adjunct clinical lecturer)

‘Through this interprofessional review of the patient case we find new stuff and actually do something good for the patient.’ (adjunct clinical lecturer)

1. Facilitating factors and challenges for the learning activity

The adjunct clinical lecturers found it difficult to get the students’ clinical supervisors to participate in the seminar because they were very busy, especially the medical supervisors. Good advance planning and several reminders were needed to engage them.

The logistics of the activity required a lot of planning, but sharing the work between several people made it easier. The activity became smoother each time it was repeated. The information documents provided by the project team greatly facilitated the process. According to the interviewees, the patient had to be chosen with care in order to make the activity rewarding for all students. Even the patient should find it pleasant to participate.

Both planning and implementation required extra time compared to individual student activities, and it was feared that implementation of the activity after the end of the project might be difficult without extra financial compensation.

‘When we had done it [prepared the activity] a couple of times … we became more experienced and it worked smoother and faster.’ (supervisor)

‘Finding a suitable patient [for the home visit]. I think, that was the hardest part.’ (supervisor)

1. Organisational prerequisites for IPL and teamwork in primary healthcare

The interviewees reported that it was of great significance for interprofessional collaboration whether clinics belonged to the same or different organisations. Geographic proximity or distance was also perceived as an important factor.

Many of the supervisors were severely pressured by high requirements in their clinical work and had difficulty setting aside the needed time. The financial compensation for supervision of students was reported not to cover the revenue loss for the unit because the time devoted for supervision led to fewer patient visits.

The operations manager at the healthcare unit was seen as an important promoter for interprofessional collaboration in clinical work. If they supported this kind of collaboration that could be more important than participating in this project.

It was difficult to organise IPL activities when students from the different study programmes did their clinical placements in primary healthcare at different time periods. The interviewees thought that increased cooperation with the study programmes would facilitate the planning of the IPL activities. The intended learning outcomes for IPL described in the different syllabi could also be further emphasised. The adjunct clinical lecturers thought that the supervisors would also need more information about IPL from the health education programmes.

Interprofessional collaboration in primary healthcare clinics was described to vary between different units and was not so common in primary healthcare centres. The supervisors found it frustrating that the home visit did not correspond with how they worked in reality. They perceived it as a weakness that they did not work in the same way as they taught, e.g. team-based.

‘Financially it doesn’t pay to have students. Nevertheless, it [supervision] is very important.’ (supervisor)

‘It has been great fun and very rewarding, but the [lack of] time and the pressure from all directions …’ (supervisor)

‘It could work much better if we could negotiate some kind of cooperation with the university colleges.’ (adjunct clinical lecturer)

1. Opportunities for dissemination

The adjunct clinical lecturers and supervisors did not see realistic opportunities to offer all students this form of learning activity in its current format. Instead, simpler IPL activities were suggested that do not require as much time for preparation and performance.

More supervisors need to be involved to disseminate the learning activity to other units in order to benefit more students. The adjunct clinical lecturers also believed the positive aspects of IPL activities could be spread as more units and people become involved.

The interviewees felt that they got to know each other better through the project, which made them more open to further interprofessional collaboration. Some units reported that they had increased their collaboration with each other and that interprofessional home visits had become more common in their daily clinical work.

The support from adjunct clinical lecturers was perceived as crucial during the project. Further support was seen as important even after the closure of the project in order for the IPL activity to be continued in the future.

‘Later, outside this project, we will have to arrange many more [interprofessional] activities, and then we have to calm down a little bit…. It should not involve that much preparation, just let the students come along’ (adjunct clinical lecturer)

‘We can see how much you gain by working together. I reckon that we are actually doing it more often now than we used to before [the project].’ (supervisor)

‘A lot of it is thanks to the adjunct clinical lecturers because we had their help and support.’ (supervisor)

### Interrelationship between quantitative and qualitative data

Data gathered via student questionnaires and in the interviews came from different respondents. The results from the analyses of these data showed alignment and they confirmed each other, e.g. students gained an increased insight into each other’s professional competencies.

## Discussion

This study evaluated patient-centred home visits as an educational model for IPL in primary healthcare. The evaluation found positive results overall, but there were some challenges. The students reported that they had gained insight into how different professions could collaborate, as well as an increased understanding of teamwork and of the other professions’ areas of competence. All patients were satisfied with the visits and felt that they had been listened to and treated with respect by the students. The analysis of the interviews with adjunct clinical lecturers and supervisors identified one overarching theme: ‘*Interprofessional home visits in primary healthcare were an appreciated and effective pedagogical learning activity with a sustainability dependent on organisational factors’*.

Participating in interprofessional shared tasks including discussions and reflections enhanced teamwork skills according to a review by Kent *et al*. (2017), which is in line with our results, even though the IPL activity in our study lasted only for three hours.

The IPL activity was organised in a way that gave students the freedom and responsibility to decide both how to perform the home visit and how to collaborate afterwards when working on the care plan for the patient. The supervisors and adjunct clinical lecturers’ reports showed that student engagement in the tasks was high. Gudmundsen *et al*. (2018) observed similar effects on mutual engagement when they let students shape their collaborative practice on clinical placements in a primary care setting.

It has previously been found that students are likely to have preconceptions about other professions (Tunstall-Pedoe *et al*., 2003); however, participating in IPL activities increases their knowledge and understanding of other students’ professional roles and practices (Kent *et al.*, 2017; Mahler *et al.*, 2018), a finding that was confirmed in the present study.

Confirmation of students’ knowledge by supervisors is important in learning. All adjunct clinical lecturers expressed the importance of ensuring that all student professions’ supervisors attended the seminars after the home visits. It has been shown that interprofessional cooperation in supervision is important for successful IPL (Laksov *et al.*, 2015).

Several studies have found that interprofessional educational activities can improve students’ interprofessional competencies (Ponzer *et al.*, 2004; Hammick *et al.*, 2007; Jacobsen and Lindqvist, 2009). Brack and Shields (2019) found that activities of a short duration also contribute to preparing students for collaborative interprofessional practice. Although the activity in the present study lasted only three hours, both supervisors and adjunct clinical lecturers perceived that the students developed in their professional and interprofessional roles through participating.

Students in healthcare programmes have commonly reported that they feel it is important to learn how to work in teams (Morison *et al.*, 2004; Mahler *et al.*, 2018). However, medical students tend to have a more negative attitude towards IPL than nursing students, and they also tend to be more protective of their own professional learning (Morison *et al.*, 2004). The medical students in the present study had very short clinical placements, typically one week long, which might have affected their responses. For instance, they may have experienced stress and concerns about reaching their own professional learning goals in such a short clinical placement (Tran *et al*., 2018). Furthermore, most of the medical students were male, and previous studies have found that male students hold less positive attitudes towards IPL than female students (Reynolds, 2003; Pollard *et al*., 2005). In a study by Mahler *et al*. (2018), the students described how there were different levels of medical knowledge between students from different healthcare professions. This might help to explain the finding in the present study where medical and nursing students more commonly reported that they felt that the patient’s needs for medical care had not been fully noted.

The supervisors who participated in the home visits and seminars also reported that they found the activity to be rewarding, and some even expressed that they had learned from students and supervisors from other professions. This is in line with a study by Attrill, Brebner and Marsh (2018) where facilitators for interprofessional clinical placements perceived that they learned from working together with students.

According to the interviewed clinicians, students also contributed to the patient care by revealing new information about the patient during the home visits. Kent and Keating (2013) reported similar findings in their study, where an interprofessional group of students reviewed elderly patients’ health needs. They also found that patients were positive towards the intervention, which is in line with our results. Studies have shown that patient satisfaction with interprofessional student teams is usually high (Kent *et al.*, 2017; Fröberg *et al.*, 2018; Oosterom *et al.*, 2019). Interaction and dialogue involving the patient, particularly where the patient can share his or her perspective, increases the recognition and awareness of the patient’s perspective and thus creates a positive learning outcome for the students (Kent *et al.*, 2017).

According to a systematic review of interprofessional education studies, organisational factors and contextual issues can play a crucial role in implementing interprofessional educational activities (Reeves *et al.*, 2016). Some of these factors were also emphasised in the interviews with adjunct clinical lecturers and supervisors in the present study. For example, the support of local leadership in terms of dedicating time for organising the activity and for supervisors to spend time in teaching was highlighted by the interviewees. Scheduling and logistics were also identified as important barriers to the implementation of interprofessional education in other studies (Abu-Rish *et al.*, 2012; de Vries-Erich *et al.*, 2017). Several interviewees in our study expressed doubts that the learning activity could be sustainable without the contribution from the adjunct clinical lecturers.

The results in this study indicate that separate organisations and geographical distances are obstacles both for interprofessional collaboration and for IPL. However, it is equally important to recognise that effective collaboration does not automatically emerge when different healthcare professions are brought together in teams or are working in the same premises. In reality, this must be trained and practiced: teamwork builds on a knowledge of each other’s competencies, and a mutual trust must develop before collaborative processes can be established (D’Amour *et al.*, 2005). Indeed, there are a wide range of human dynamics that need to develop within a team (D’Amour *et al.*, 2005). Barriers to collaboration have previously been described and can result in poor teamwork. Examples include working in silos, a hierarchical culture, and a profession-centred rather than person-centred approach (Meisinger and Wohler, 2016; Zaudke *et al.*, 2016).

Several previous studies have shown that well-functioning interprofessional teams lead to improvements in quality of care, patient satisfaction, patient safety, and job satisfaction (Gilbert *et al*., 2010; Petri, 2010). Further, well-functioning interprofessional teams have also been found to reduce stress among healthcare professionals and to reduce costs (Gilbert *et al*., 2010; Petri, 2010). For effective interprofessional collaboration, there also needs to be awareness and competence in person-centred care, communication, role distribution within the team, conflict management, and team spirit (Drummond *et al.*, 2012).

A supportive leadership focused on collaboration has previously been shown to facilitate interprofessional collaboration (Drummond *et al.*, 2012). This was also highlighted in the current study as a critical factor for enabling the organisation of the learning activity. In order to work in efficient interprofessional teams, management support is required. The healthcare system therefore needs to produce clear policy documents that govern and demonstrate the process behind these ways of working. Further, the healthcare system should endeavour to create appropriate conditions through contracts and functional reimbursement systems (Oandasan and Reeves, 2005).

Another obstacle was that students’ periods of clinical training in primary care were not consistent between different healthcare programmes, which led to practical difficulties in administering IPL. A closer cooperation and co-planning with health education programmes would make the implementation of IPL activities easier and might increase their sustainability. Important success factors in the Leicester model include close collaboration among different education programmes and embedding the course within curricula (Anderson and Lennox 2009). The establishment of planning groups with representatives from the educational institutions, clinical settings, and other relevant external stakeholders is important for implementing interprofessional education (Mayall *et al.*, 2004). Administrative support and financial factors are also crucial (Anderson & Lennox 2009; Pottie *et al.*, 2009; Paquette-Warren *et al.*, 2014).

Introducing IPL activities in primary healthcare settings is challenging (Miller *et al.*, 2019). In the present study, the experience of interprofessional collaboration and IPL varied between adjunct clinical lecturers and supervisors, and most of them had little experience with interprofessional collaboration in their clinical everyday work. With that background, it can be difficult for supervisors to initiate a model that they are not entirely comfortable with. However, the supervisors involved in the project had the opportunity to increase their expertise in interprofessional collaboration, which made them more confident in their role and contributed to increased quality of care. They also obtained knowledge through interacting with the other supervisors, and especially from the adjunct clinical lecturers who participated in the organisation of the project and the seminars. The increased knowledge and experience of the supervisors could in the long term lead to a better learning environment for future students who may have the opportunity to participate in a natural interprofessional collaboration in their daily work during their clinical training. This concept is supported by earlier work that has shown that interprofessional education can be facilitated by an established collaboration between educational institutions, clinical practices, and senior leaders (Price *et al.*, 2009; Meisinger and Wohler, 2016).

### Strengths and limitations

This study was performed in six different geographical areas in Stockholm primary healthcare, which ensured a broad and varied data sampling. Healthcare students were randomly allocated to the participating primary healthcare clinics from which students were invited to participate in home visits. However, the number of participating students was still limited, and many students in primary health care did not get the opportunity to participate. All participation by clinics, students, and supervisors was voluntary, with a risk for selection bias, so the results may not completely reflect how a broad implementation of such an activity would work in the entirety of primary health care. Participating students, supervisors, and adjunct clinical lecturers represented many different professions in primary health care, and this gave greater weight to the data, which were analysed from the perspective of different professions. A total of 30 home visits were performed in which 109 students participated. This means that the sampled data captured a large number of experiences. The data comprised all participants’ perceptions (students, patients, adjunct clinical lecturers, and supervisors), which gave a comprehensive picture of how this activity might contribute to students’ IPL, and also provided a broad perspective on how such an activity might be implemented and performed. The uneven gender distribution among students, supervisors, and adjunct clinical lecturers may act as a limitation. Most of the medical students were male, which does not reflect the gender distribution in medical programmes in Sweden today where there are slightly more female than male students. We do not know, though, whether this had any influence on our results. The sex distribution among other than medical students was representative of the proportions of different sexes in their respective educational programmes. Some professions had low representation among the students, which might have limited our possibility to describe their experiences fully. The researchers and the participants (students, adjunct clinical lecturers, and supervisors) represented different professions, which strengthens the credibility of the results. The transferability of the results is somewhat limited because of the study being relatively narrow regarding numbers of participants and geographical location, but primary healthcare has similar obstacles in several countries in the field of IPL. Consequently, we believe that some of our conclusions are relevant for other primary healthcare settings.

## Conclusions

The students felt that participation in the interprofessional home visits increased their understanding of collaboration and of other professions’ skills. The patients were positive towards the activity, and the supervisors found the home visits to be an appreciated and effective learning activity. However, there were barriers for implementing home visits in the context of the reimbursement system and the high clinical workload of the supervisors. Interprofessional collaboration was not routine in the supervisors’ daily clinical work. The results of the study indicate that the learning activity can be used in primary healthcare settings to promote students’ IPL but that organisational factors need to be considered in order to support sustainability.

## References

[ref1] Abu-Rish E , Kim S , Choe L , Varpio L , Malik E , White AA , Craddick K , Blondon K , Robins L , Nagasawa P , Thigpen A , Chen LL , Rich J and Zierler B (2012) Current trends in interprofessional education of health sciences students: a literature review. Journal of Interprofessional Care 26(6), 444–451. 22924872 10.3109/13561820.2012.715604 PMC7594101

[ref2] Anderson ES and Lennox A (2009) The Leicester model of interprofessional education: developing, delivering and learning from student voices for 10 years. Journal of Interprofessional Care 23(6), 557–573. 19842950 10.3109/13561820903051451

[ref3] Attrill S , Brebner C and Marsh C (2018) Learning from students: facilitators’ learning in interprofessional placements. Journal of Interprofessional Care 32(5), 603–612. 29746192 10.1080/13561820.2018.1470497

[ref4] Bodenheimer T , Wagner EH and Grumbach K (2002) Improving primary care for patients with chronic illness. JAMA 288(14), 1775–1779. 12365965 10.1001/jama.288.14.1775

[ref5] Brack P and Shields N (2019) Short duration clinically-based interprofessional shadowing and patient review activities may have a role in preparing health professional students to practice collaboratively: a systematic literature review. Journal of Interprofessional Care 33(5), 446–455. 30395747 10.1080/13561820.2018.1543256

[ref6] CAIPE, The Centre for the Advancement of Interprofessional Education (2002) *Interprofessional education-a definition* Retrieved from www.caipe.org.uk

[ref7] D’Amour D , Ferrada-Videla M , San Martin Rodriguez L and Beaulieu MD (2005) The conceptual basis for interprofessional collaboration: core concepts and theoretical frameworks. Journal of Interprofessional Care 19(Suppl 1), 116–131. 16096150 10.1080/13561820500082529

[ref8] de Vries-Erich J , Reuchlin K , de Maaijer P and van de Ridder JM (2017) Identifying facilitators and barriers for implementation of interprofessional education: perspectives from medical educators in the Netherlands. Journal of Interprofessional Care 31(2), 170–174. 28181853 10.1080/13561820.2016.1261099

[ref9] Drummond N , Abbott K , Williamson T and Somji B (2012) Interprofessional primary care in academic family medicine clinics: implications for education and training. Canadian Family Physician 58(8), e450–e458. 22893347 PMC3419002

[ref10] Fröberg M , Leanderson C , Fläckman B , Hedman-Lagerlöf E , Björklund K , Nilsson GH and Stenfors T (2018) Experiences of a student-run clinic in primary care: a mixed-method study with students, patients and supervisors. Scandinavian Journal of Primary Health Care 36(1), 36–46. 29368978 10.1080/02813432.2018.1426143 PMC5901439

[ref11] Gilbert JH , Yan J and Hoffman SJ (2010) A WHO report: framework for action on interprofessional education and collaborative practice. Journal of Allied Health 39(Suppl 1), 196–197. 21174039

[ref12] Graneheim UH and Lundman B (2004) Qualitative content analysis in nursing research: concepts, procedures and measures to achieve trustworthiness. Nurse Education Today 24(2), 105–112. 14769454 10.1016/j.nedt.2003.10.001

[ref13] Gudmundsen AC , Norbye B , Abrandt Dahlgren M and Obstfelder A (2018) Interprofessional student meetings in municipal health service – Mutual learning towards a Community of Practice in patient care. Journal of Interprofessional Care, 1–9. 10.1080/13561820.2018.1515732 30207498

[ref14] Hammick M , Freeth D , Koppel I , Reeves S and Barr H (2007) A best evidence systematic review of interprofessional education: BEME Guide no. 9. Medical Teacher 29(8), 735–751. 18236271 10.1080/01421590701682576

[ref15] Jacobsen F and Lindqvist S (2009) A two-week stay in an Interprofessional Training Unit changes students’ attitudes to health professionals. Journal of Interprofessional Care 23(3), 242–250. 19280378 10.1080/13561820902739858

[ref16] Kent F , Hayes J , Glass S and Rees CE (2017) Pre-registration interprofessional clinical education in the workplace: a realist review. Medical Education 51(9), 903–917. 28612407 10.1111/medu.13346

[ref17] Kent F and Keating J (2013) Patient outcomes from a student-led interprofessional clinic in primary care. Journal of Interprofessional Care 27(4), 336–338. 23421346 10.3109/13561820.2013.767226

[ref18] Laksov KB , Boman LE , Liljedahl M and Björck E (2015) Identifying keys to success in clinical learning: a study of two interprofessional learning environments. Journal of Interprofessional Care 29(2), 156–158. 25070425 10.3109/13561820.2014.942777

[ref19] Mahler C , Schwarzbeck V , Mink J and Goetz K (2018) Students´ perception of interprofessional education in the bachelor programme “Interprofessional Health Care” in Heidelberg, Germany: an exploratory case study. BMC Medical Educaion 18(1), 19. 10.1186/s12909-018-1124-3 PMC5785847 29370784

[ref20] Mayall E , Oathamshaw S , Lovell K and Pusey H (2004) Development and piloting of a multidisciplinary training course for detecting and managing depression in the older person. Journal of Psychiatric and Mental Health Nursing 11(2), 165–171. 15009491 10.1111/j.1365-2850.2003.00702.x

[ref21] Meisinger K and Wohler D (2016) Walking the walk in team-based education: the crimson care collaborative clinic in family medicine. AMA Journal of Ethics 18(9), 910–916. 27669136 10.1001/journalofethics.2016.18.9.medu1-1609

[ref22] Miller R , Scherpbier N , van Amsterdam L , Guedes V and Pype P (2019) Inter-professional education and primary care: EFPC position paper. Primary Health Care Research & Development 20, e138. 31581968 10.1017/S1463423619000653 PMC6784359

[ref23] Morison S , Boohan M , Moutray M and Jenkins J (2004) Developing pre-qualification inter-professional education for nursing and medical students: sampling student attitudes to guide development. Nurse Education in Practice 4(1), 20–29. 19038133 10.1016/S1471-5953(03)00015-5

[ref24] Oandasan I and Reeves S (2005) Key elements of interprofessional education. Part 2: factors, processes and outcomes. Journal of Interprofessional Care 19(Suppl 1), 39–48. 16096144 10.1080/13561820500081703

[ref25] Oosterom N , Floren LC , Ten Cate O and Westerveld HE (2019) A review of interprofessional training wards: enhancing student learning and patient outcomes. Medical Teacher 41(5), 547–554. 30394168 10.1080/0142159X.2018.1503410

[ref26] Paquette-Warren J , Roberts SE , Fournie M , Tyler M , Brown J and Harris S (2014) Improving chronic care through continuing education of interprofessional primary healthcare teams: a process evaluation. Journal of Interprofessional Care 28(3), 232–238. 24397571 10.3109/13561820.2013.874981 PMC4025597

[ref27] Petri L (2010) Concept analysis of interdisciplinary collaboration’. Nursing Forum 45(2), 73–82. 20536755 10.1111/j.1744-6198.2010.00167.x

[ref28] Pollard K , Miers ME and Gilchrist M (2005) Second year scepticism: pre-qualifying health and social care students’ midpoint self-assessment, attitudes and perceptions concerning interprofessional learning and working. Journal of Interprofessional Care 19(3), 251–268. 16029979 10.1080/13561820400024225

[ref29] Ponzer S , Hylin U , Kusoffsky A , Lauffs M , Lonka K , Mattiasson AC and Nordström G (2004) Interprofessional training in the context of clinical practice: goals and students’ perceptions on clinical education wards. Medical Education 38(7), 727–736. 15200397 10.1111/j.1365-2929.2004.01848.x

[ref30] Pottie K , Haydt S , Farrell B , Kennie N , Sellors C , Martin C , Dolovich L and IMPACT team members (2009) Pharmacist’s identity development within multidisciplinary primary health care teams in Ontario; qualitative results from the IMPACT project. Research in Social and Administrative Pharmacy 5(4), 319–326. 19962675 10.1016/j.sapharm.2008.12.002

[ref31] Price D , Howard M , Hilts L , Dolovich L , McCarthy L , Walsh AE and Dykeman L (2009) Interprofessional education in academic family medicine teaching units: a functional program and culture. Canadian Family Physician 55(9), 901-1.e1-5. PMC2743589 19752260

[ref32] Reeves S , Fletcher S , Barr H , Birch I , Boet S , Davies N , McFadyen A , Rivera J and Kitto S (2016) A BEME systematic review of the effects of interprofessional education: BEME Guide No. 39. Medical Teacher 38(7), 656–668. 27146438 10.3109/0142159X.2016.1173663

[ref33] Reynolds F (2003) Initial experiences of interprofessional problem-based learning: a comparison of male and female students’ views. Journal of Interprofessional Care 17(1), 35–44. 12772468 10.1080/1356182021000044148

[ref34] Thistlethwaite J (2012) Interprofessional education: a review of context, learning and the research agenda. Medical Education 46(1), 58–70. 22150197 10.1111/j.1365-2923.2011.04143.x

[ref35] Tran C , Kaila P and Salminen H (2018) Conditions for interprofessional education for students in primary healthcare: a qualitative study. BMC Medical Education 18(1), 122. 29866079 10.1186/s12909-018-1245-8 PMC5987484

[ref36] Tunstall-Pedoe S , Rink E and Hilton S (2003) Student attitudes to undergraduate interprofessional education. Journal of Interprofessional Care 17(2), 161–172. 12745298 10.1080/1356182031000081768

[ref37] Zaudke JK , Paolo A , Kleoppel J , Phillips C and Shrader S (2016) The impact of an interprofessional practice experience on readiness for interprofessional learning. Family Medicine 48(5), 371–376. 27159096

